# Prescription Patterns of Sacubitril/Valsartan in an Outpatient Population Diagnosed with Heart Failure with Reduced Ejection Fraction After a Recent Hospitalization

**DOI:** 10.3390/epidemiologia6030055

**Published:** 2025-09-05

**Authors:** Dimitri Roustan, Hugo Bothorel, Omar Kherad

**Affiliations:** 1 Division of Emergency Medicine, Cliniques Universitaires Saint-Luc, 1200 Bruxelles, Belgium; dimitri.roustan@saintluc.uclouvain.be; 2 Research Unit, Hôpital de La Tour and University of Geneva, Avenue J.-D.-Maillard 3, 1217 Geneva, Switzerland; 3 Division of Internal Medicine, Hôpital de La Tour and University of Geneva, Avenue J.-D.-Maillard 3, 1217 Geneva, Switzerland; 4 Choosing Wisely Working Group at the European Federation of Internal Medicine, Leerlooierijstraat 18/004, 1930 Zaventem, Belgium

**Keywords:** medication overuse, medication underuse, healthcare efficiency, guideline-directed medical therapy

## Abstract

**Background**: Sacubitril/Valsartan is a first-line treatment for heart failure with reduced ejection fraction (HFrEF) according to international guidelines. However, achieving the target doses of guideline-directed medical therapy (GDMT) remains a challenge in clinical practice and its efficacy at suboptimal dose (<200 mg/day) versus angiotensin-converting enzyme (ACE) inhibitors remains debated. Our objective was to evaluate the titration of Sacubitril/Valsartan within 3 months of hospital discharge in patients with HFrEF. **Methods**: A cross-sectional study was conducted in a secondary care hospital in Geneva, Switzerland. Patients hospitalized between 2020 and 2022 with HFrEF, discharged with Sacubitril/Valsartan, were included. Physicians managing patients discharged with a Sacubitril/Valsartan dose of less than 200 mg/day were contacted and asked to complete a structured 7-item questionnaire regarding dose adjustments within the first 3 months following hospital discharge. The primary outcome was the proportion of patients who did not achieve GDMT doses of Sacubitril/Valsartan, along with reasons for inadequate titration. **Results**: Overall, 30 patients out of 79 (38%, 95% confidence interval [27–49%]) had not been titrated to an effective dose of Sacubitril/Valsartan 3 months after hospitalization. Of these thirty patients, the primary reason for not titrating cited by their practitioners (*n* = 27) was that titration was perceived to be within the cardiologist’s scope of responsibility (15/27, 56%). While most physicians (66%) knew the target doses for Sacubitril/Valsartan, 83% of them were unaware that the clinical benefit of sacubitril/valsartan at doses below 50% of the target compared to ACE inhibitors remains uncertain and is not well supported by current evidence. **Conclusions**: In this cohort, more than a third of patients with HFrEF were not titrated to guideline-recommended target doses of sacubitril/valsartan within 3 months of hospital discharge. This finding raises questions about the clinical and economic value of initiating sacubitril/valsartan without subsequent dose optimization, especially given the uncertainty surrounding the efficacy of suboptimal dosing compared to ACE inhibitors.

## 1. Background

Heart failure represents a major and escalating global public health burden, affecting over 64 million individuals worldwide [1]. It is a clinical syndrome defined by characteristic symptoms and signs resulting from structural and/or functional cardiac abnormalities. Classification is primarily based on left ventricular ejection fraction (LVEF), distinguishing heart failure with reduced ejection fraction (HFrEF), mildly reduced ejection fraction (HFmrEF), and preserved ejection fraction (HFpEF). Despite therapeutic advances, it continues to be a leading cause of morbidity and mortality, placing substantial strain on healthcare systems through frequent hospitalizations and elevated readmission rates. Evidence-based management is pivotal to improving outcomes in patients with heart failure, with the initiation and uptitration of guideline-directed medical therapy (GDMT) constituting its cornerstone. In HFrEF, GDMT encompasses four foundational pharmacological classes: ACE inhibitors (ACE-I) (interchangeable with angiotensin receptor blockers) or angiotensin receptor-neprilysin inhibitors (ARNIs), beta-blockers, mineralocorticoid receptor antagonists (MRAs) and sodium-glucose 2 transporter (SGLT2) inhibitors. Each of these classes has demonstrated efficacy in reducing all-cause mortality, improving symptoms, and decreasing the risk of heart failure-related hospitalization [2]. Nevertheless, despite robust guideline recommendations, a substantial proportion of patients with HFrEF remains undertreated with GDMT [3]. Challenges in achieving optimal therapy include uncertainties about the sequencing of therapies, titration timeframes, and the management of medication side effects and adherence.

Sacubitril/valsartan (S/V), a combination of a neprilysin-inhibitor and an angiotensin II receptor blocker (ARNI), was shown to reduce cardiovascular mortality and heart failure hospitalizations compared to enalapril, as demonstrated in the landmark PARADIGM-HF trial [4]. Subsequent trials often started with lower initial doses of 50–100 mg/day, with a target dose of 400 mg/day achieved through titration over 3 to 6 weeks [5,6]. These positive results prompted the incorporation of ARNIs into heart failure guidelines as a first-line treatment for HFrEF. However, a post hoc analysis of PARADIGM-HF revealed that the effect of S/V versus enalapril at doses below 50% of the target remains uncertain with current evidence providing only limited support [7]. Some clinical trials have indicated that a substantial proportion of patients are not titrated to optimal doses [5,8], thus potentially limiting clinical efficacy and increasing costs without demonstrated benefit. Therefore, the effects of low-dose versus high-dose S/V in patients with heart failure remain controversial, potentially complicating clinical decision-making regarding the optimal S/V dosing strategy for effective HFrEF management.

To assess the gap between clinical practice and guideline recommendations, we conducted a local study to evaluate the proportion of HFrEF patients who did not achieve GDMT doses of S/V within three months of discharge from our hospital. Additionally, we sought to identify potential barriers to effective dose titration and to initiate a discussion on the cost-effectiveness of this high-cost treatment when prescribed at suboptimal doses.

## 2. Materials and Methods

We conducted a retrospective, cross-sectional, single-center study in a secondary care hospital (La Tour Hospital) in Switzerland. La Tour Hospital is a private hospital with 180 beds. The internal medicine residency program is academically affiliated with the University of Geneva, enrolling 23 residents, 2 junior staff and 6 senior staff. The internal medicine division contains 64 acute care hospital beds distributed among 4 clinical teaching units. Patient data were collected from the institution’s Electronic Medical Record (Carefolio, Tecost SA, Fribourg, Switzerland).

Adult patients more than 18 years of age, admitted to our institution between January 2020 and December 2022, with a chronic condition of HFrEF and a discharge prescription for S/V were included. We then divided these patients into two groups; the first group comprised patients with a discharge dose of S/V ≥ 200 mg per day, and a second group discharged with doses below 200 mg per day. No distinction was made between hospital-initiated and outpatient-initiated S/V prescriptions. Based on the post hoc analysis of PARADIGM-HF, we defined the suboptimal dose of S/V as a prescription of <200 mg/day (<48/51 twice a day) and the GDMT target dose as 400 mg/day [2,7]. Of note, the index hospitalization was not required to be related to an acute heart failure event. The study protocol was approved by the ethics committee of Geneva (CCER 2023-02006).

The methodological assumption was made that patients with an S/V discharge dose of at least 200 mg/day remained at optimal doses during the 3-month period. For the group discharged with a dose of less than 200 mg/day, considered suboptimal, we contacted each patient’s primary care physician or cardiologist, depending on who managed the initial follow-up, to determine whether the patient had been titrated to at least 50% of the predefined target dose of S/V (≥200 mg/day) within three months of the initial hospitalization.

**Figure 1:**
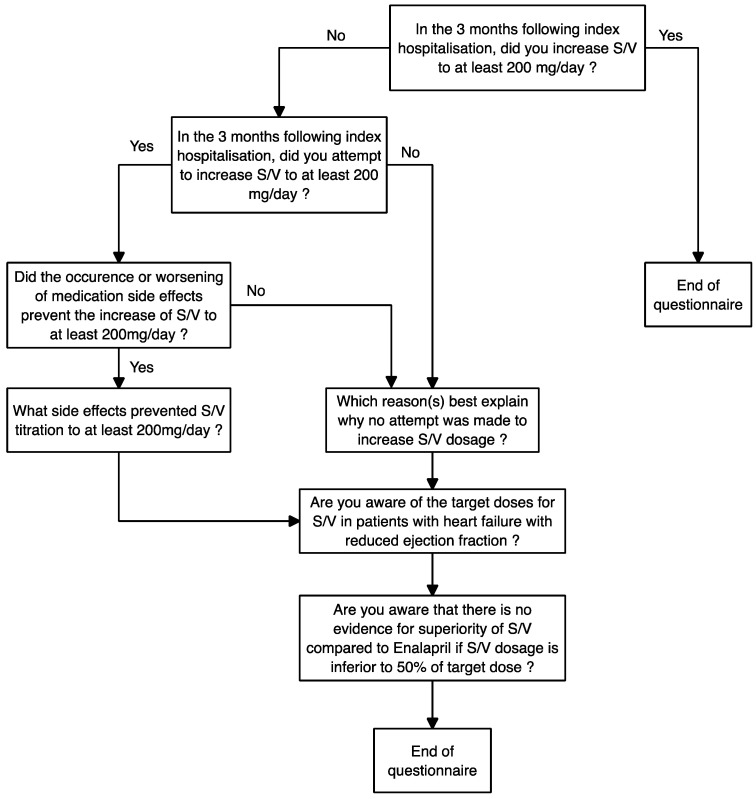
Questionnaire algorithm.

Once the physician’s consent was obtained, we conducted a focused interview using an ad hoc questionnaire as shown in Figure 1. The questionnaire was developed by the three authors through discussion and consensus on relevant clinical outcomes, followed by a rapid face validation process.

The primary outcome of this study was the percentage of patients with suboptimal doses of S/V, 3 months after hospitalization at our institution, among all patients discharged with S/V. The secondary objectives of this study were to assess the proportion of patients who remained at suboptimal S/V doses at 3 months among those initially discharged with suboptimal dosing. Additionally, a secondary qualitative objective was to identify the underlying reasons for the failure to achieve optimal S/V titration within this period.

An a priori sample size calculation was performed to ensure adequate statistical power. In a similar population, another study reported that 42% of patients received a suboptimal dose of S/V [8]. We estimated that a total sample size of 60 patients would be needed to evaluate such prevalence with a 12.5% precision and a confidence interval of 95%. A 25% margin was added to this sample size, which led to a minimum needed inclusion of 80 cases. This sample size was calculated using the following formula, where *p* is the estimated prevalence (42%), *Z* the Z-score for 95% CI (1.96) and *d* the precision (12.5%):

$$Sample\;size=\frac{Z^{2}P(1-P)}{d^{2}}$$

The normality of continuous variable distributions was assessed and determined using a histogram, QQ plot and Shapiro–Wilk test. For baseline characteristics, continuous data was reported as mean ± standard deviation (for normally distributed data) or median [Interquartile range (IQR)] while categorical data was reported as proportions. The proportion of patients with ineffective S/V dose within three months of hospital discharge was reported with 95% confidence intervals (CI) using the following formulas, where $\hat{p}$ is the real calculated proportion, Z the Z-score for 95% CI (1.96) and *n* the number of patients in the cohort:

$$Lower\;limit=\hat{p}-Z\sqrt{\frac{\hat{p}(1-\hat{p})}{n}}$$

$$Upper\;limit=\hat{p}+Z\sqrt{\frac{\hat{p}(1-\hat{p})}{n}}$$

Statistical analyses were performed using R version 4.4.2 (R Foundation for Statistical Computing, Vienna, Austria).

## 3. Results

**Figure 2:**
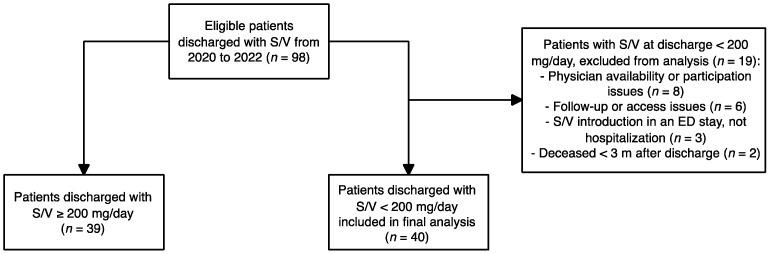
Patient selection.

From January 2020 to December 2022, 98 patients had a prescription of S/V at discharge from our institution (including hospitalization and ED discharge). At discharge, 39 patients (40%) had an S/V dose of at least 200 mg/day while 59 (60%) had a suboptimal S/V dose (<200 mg/day). Within the suboptimal dosage group, 19 patients were excluded from the final analysis (9 for physician availability or participation issues, 6 for issues related to follow-up, 2 due to introduction of S/V during an ED stay without hospitalization, and 2 due to being deceased before the 3-month time period). After these exclusions, a total of 79 patients with 3-month S/V dosage information were included in the final analysis (Figure 2). A total of 35 physicians conducted the ambulatory follow-up for patients with suboptimal S/V at discharge, with some physicians managing more than one patient in the study population (Table 1).

The median age of the study population was 77 years, with a median left ventricular ejection fraction (LVEF) of 30%. Most patients were overweight, with a median body mass index (BMI) of 28.9 kg/m^2^. Documented coronary ischemia and arterial hypertension were present in 62.5% and 52.5% of patients, respectively. Beta-blockers and diuretics were prescribed in 88% and 75% of cases, respectively. Follow-up was predominantly conducted by general practitioners (72.5%).

Overall, at least 30 patients out of the 79 eligible (38% [95% CI, 27–49%]) had not been titrated to an optimal dose of S/V (at least 200 mg/day) within three months of hospitalization. In 27 patients, no dose titration was attempted, whereas in the remaining three cases, titration was not pursued due to reported adverse effects. The primary reason cited by general practitioners for not titrating the dose was the perception that this responsibility lay within the cardiologist’s scope of practice. (15/27, 56%) (Table 2). While most physicians were aware of the target doses for S/V (23/35, 66%), only a minority (6/35, 17%) recognized that the clinical benefit of S/V at doses below 50% of the target, relative to ACE inhibitors, remains uncertain and is insufficiently supported by current evidence.

## 4. Discussion

In this local, real-life study, we found that at least 38% of patients with HFrEF were not titrated to minimally effective doses of S/V at 3 months post-discharge from a secondary care hospital, confirming that it may be challenging to achieve target doses of S/V in everyday clinical practice [9]. This finding is consistent with a recent trial in patients with HFmrEF and HFrEF, in which 42% of participants were not titrated to a minimum of 200 mg daily [8]. Similarly, in a trial assessing S/V titration tolerability in HFrEF patients, at least 19% were unable to tolerate the target dose of 200 mg twice daily during the whole duration of the study (12 weeks) [5]. Furthermore, 75% of patients that were already discharged from our institution with a suboptimal S/V dose remained inadequately titrated at 3 months.

These results highlight converging gaps in the implementation of GDMT. Notably, only a minority of physicians in our study were aware of the limited and somewhat inconclusive evidence regarding differences in clinical benefit between sacubitril/valsartan (S/V) and enalapril at doses below 50% of the target in the post hoc analysis of PARADIGM-HF. This uncertainty may partially explain why most patients in our cohort were not uptitrated beyond 200 mg of S/V daily. Additionally, over half of the physicians interviewed perceived S/V titration as primarily the responsibility of the cardiologist, indicating a modifiable barrier to optimal GDMT optimization. This perception points to a clear opportunity to improve titration practices. Targeted heart failure education and the empowerment of primary care physicians represent promising strategies to enhance medication titration. Moreover, when combined with predefined titration protocols, structured follow-up within multidisciplinary heart failure teams has demonstrated improved attainment of target doses without increased adverse events [10,11]. Based on the existing recommendations, we propose an algorithm for uptitration and follow-up of these patients (Supplementary Material—Figure S1. Sacubitril/Valsartan titration algorithm Flowchart.).

The final doses achieved in real-world practice are often substantially lower than those attained in PARADIGM-HF, where elderly and frail patients were underrepresented, even if the beneficial effects of S/V on the primary composite outcome were independent of baseline frailty class [12]. Differences in patient profiles encountered in clinical practice might lead to hesitation for initiating or uptitrating S/V, as frail patients are more likely to experience polypharmacy, drug interactions, adverse drug reactions, and treatment discontinuation. Adverse events, although more frequent in frail patients overall, were not more common among those randomized to S/V versus valsartan in an HFpEF population [13]. These findings suggest that the risk–benefit balance of S/V in frail patients may be more favorable than previously assumed, challenging the common reluctance of clinicians to uptitrate S/V due to doubts about the risk-benefit balance in this population. This information is especially relevant in our local study, given the higher average age compared to PARADGIM-HF (74.6 vs. 63.8 years). In addition to advanced age, other factors associated with challenges in titrating sacubitril/valsartan to target doses include lower baseline systolic blood pressure and elevated serum creatinine levels [14].

The conclusions of PARADIGM-HF have not been universally accepted, with some authors raising methodological concerns, particularly regarding dosing differences. This namely involves the administration of 400 mg/day of S/V (equivalent to 205.6 mg of valsartan) compared to 10 mg of enalapril twice daily, which represents half the recommended maximum dose outlined in the ESC and AHA heart failure guidelines [2,15]. Furthermore, all selected patients in the PARADIGM-HF trial were required to enter a single-blind prerandomization run-in phase to ensure they tolerated a prespecified maximum dose of S/V. As a result, a large proportion of patients (74.76%) achieved the target dose of S/V in the trial. Finally, a large subsequent trial comparing equivalent doses of S/V to ACE inhibitors or angiotensin receptor blockers (ARBs) showed no differences in mortality or heart failure hospitalization after myocardial infarction [6], a finding confirmed by a recent meta-analysis [16].

Thus, the minimally effective dose of S/V in comparison to ACE inhibitors remains a topic of ongoing debate. A recent meta-analysis revealed that low-dose S/V was associated with increased risks of HF hospitalization and all-cause mortality compared to high-dose S/V. Importantly, there were no significant differences between the two dosing regimens regarding improvements in NYHA classification, changes in left ventricular ejection fraction (LVEF), NT-proBNP levels, or systolic blood pressure [17]. These data demonstrate that the clinical benefits of low-dose S/V are inferior to those observed with high-dose S/V. A post hoc analysis of the PARADIGM-HF trial further suggests, to a certain extent, that lower doses of S/V can reduce heart failure-related mortality and morbidity compared to equivalent doses of ACE inhibitors, as it did not demonstrate a significant heterogeneity of treatment effect by dose. However, the absence of a statistical interaction does not equate to evidence of equivalent efficacy at lower doses, particularly given the analysis’s limited power and wide confidence intervals [7]. Indeed, the same analysis indicates that the benefits of S/V compared to ACE inhibitors are less clearly established at doses below 200 mg per day (<50% of the target), which raises concerns about lower clinical efficacy and increased costs in patients who are not uptitrated to at least 200 mg per day.

From a mechanistic perspective, the single-arm PROVE-HF trial investigated the progression of biological and echocardiographic markers of cardiac remodeling, including in patients who did not reach target doses of S/V. The study demonstrated that, even within this subgroup, S/V exerted a beneficial effect on cardiac remodeling at 12 months [18]. More recently, a post hoc analysis of the same trial demonstrated that patients taking S/V for HFrEF exhibited comparable biomarker, health status, and echocardiographic improvements across tertiles of dosing: low-dose (average daily dose 112 mg), moderate dose (342 mg), or high-dose (379 mg) [19]. However, since this study was not designed to evaluate survival, mortality outcomes according to S/V dose tertile were not reported.

Although we employed a threshold of S/V doses below 200 mg/day as a dichotomous cutoff for suboptimal therapy, the evidence discussed above indicates that S/V confers benefits along a more nuanced, continuous dose–response spectrum. This continuous relationship is supported by data from cohort studies and heart failure registries. A retrospective patient cohort demonstrated a dose-dependent incremental benefit of S/V, with doses ranging from 50 mg to 200 mg per day, on both heart failure hospitalization and all-cause mortality [20]. This dose–response relationship has also been observed in the Japanese REVIEW-HF registry [21] and a US claims-based registry [22]. Data from a Korean registry integrating mechanistic and clinical outcome assessments further demonstrated a positive dose–response relationship between low-dose and intermediate-to-high-dose S/V groups [23]. Taken together, these data suggest that S/V may confer incremental reductions in heart failure hospitalizations and all-cause mortality across the full dose range, consistent with, though not definitively establishing, a continuous rather than strictly dichotomous dose–response relationship.

These findings highlight the importance of considering achieved drug doses when interpreting clinical outcomes. The significant proportion of patients unable to achieve target doses in real-world settings emphasizes the importance of conducting further clinical trials to evaluate the outcomes of S/V compared to ACE inhibitors and ARBs at equivalent doses in patients who cannot tolerate higher doses of S/V. In this specific patient population, this research could provide evidence on the clinical and cost-effectiveness of S/V, particularly given its substantially higher cost compared to ACE inhibitors and ARBs (dose-equivalent difference ranging from 4 to 6 times the price). Indeed, medico-economic analyses have reported that the incremental cost-effectiveness ratio (ICER) per quality-adjusted life year (QALY) gained in the United States ranges from $35,356 to $249,111, depending on the base-case assumptions and time horizon considered [24,25]. ICERs vary substantially across countries [26], yet overall, S/V is generally considered cost-effective. However, existing analyses are based on data from the landmark S/V trial, and no medico-economic, head-to-head comparison with ACE inhibitors at suboptimal doses has been conducted To date, the only study stratifying S/V doses in relation to costs is a US claims-based study [22]. This study reported that total healthcare costs varied inversely with S/V dosage, a finding potentially attributable to lower all-cause hospital admission rates. Notably, cost-effectiveness considerations may become less critical once a generic formulation of S/V becomes available. In the interim, dedicated dose-stratified cost-effectiveness analyses are warranted to ensure that treatment recommendations remain patient-centered, clinically effective, and economically sustainable.

### Limitations

Our study has several limitations. First, given the high level of frontline cardiologist care in our region, generalizability of our results may not be possible in regions with differing medical demographics. Also, our study focused on S/V titration within a 3-month timeframe, which might be considered short. Nonetheless, guidelines from the ESC and ACC emphasize the importance of achieving maximally tolerated doses of GDMT within this period to optimize outcomes and inform subsequent treatment decisions [2,27]. We also made the a priori assumption that most patients with adequate S/V dosage at discharge remained at adequate doses during the 3-month period, which may have led to misclassification bias and thus skewed our results. However, this limitation would bias our findings toward underestimating (rather than increasing) the proportion of patients who failed to reach the minimum 200 mg/day S/V dose. Furthermore, a significant proportion of patients in our study were hospitalized for reasons unrelated to acute heart failure, potentially influencing the prioritization of medication titration during follow-up. Our patient population also had low SGLT-2 inhibitor use (27.5%), which may not reflect current clinical practice and could influence the titration of other GDMT medications. Also, the cross-sectional design, reliance on physician-reported outcomes and an unvalidated ad hoc questionnaire may limit generalizability and validity of our results. Concerning our questionnaire, we acknowledge several limitations. The phrasing concerning knowledge of absence of difference between S/V and ACE inhibitors may have influenced physician answers due to social-desirability bias. To minimize this risk, the questionnaire was administered by a medical resident, and participants were explicitly informed that their responses would remain anonymous and would not be used to judge their clinical practice. Furthermore, recall bias may also have influenced the physicians’ responses. For questions related to dose escalation, this risk was mitigated by referencing electronic health records and prescription histories. However, more subjective items were likely more susceptible to such bias. Finally, the limited number of events in our cohort precluded sufficient statistical power to perform more advanced analyses, such as multivariable logistic regression for assessing predictive factors of non-uptitration.

## 5. Conclusions

This local cross-sectional study confirms that at least 38% of patients discharged with suboptimal S/V doses were not titrated to optimal doses within a 3-month timeframe after hospitalization. Among those who had already been discharged with suboptimal doses of S/V, 75% were not increased to optimal doses at 3 months post-discharge. These findings highlight the need to reassess the clinical and economic value of initiating sacubitril/valsartan without subsequent dose optimization, particularly in light of the ongoing uncertainty regarding the efficacy of suboptimal dosing compared to ACE inhibitors.

## Figures and Tables

**Table 1:** Patient characteristics.

Characteristics of Patients with Suboptimal S/V at Discharge (*n* = 40)	
**Characteristics**	
Age—Median [IQR]	77 [66.8–82]
Male sex—no. (%)	31 (77%)
LVEF category	
0–9%	0
10–19%	3
20–29%	10
30–39%	17
40–49%	7
Unknown	3
LVEF—Mean ± SD	30% ± 7.9
LVEF—Median [IQR]	30% [10]
**BMI (kg/m^2^)—Median [IQR]**	28.9 [25.3–31.9]
**Comorbidities**	
Arterial Hypertension	52.5% (21/40)
Diabetes	30% (12/40)
Chronic Kidney Disease	35% (14/40)
**Atrial Fibrillation**	52.5% (21/40)
**Proven cardiac ischemia**	65% (26/40)
**Valvular anomalies**	
Aortic Stenosis	10% (4/40)
Aortic Regurgitation	30% (12/40)
Mitral Stenosis	2.5% (1/40)
Mitral Regurgitation	53% (21/40)
Tricuspid Stenosis	0% (0/40)
Tricuspid Regurgitation	38% (15/40)
Pulmonary Stenosis	0% (0/40)
Pulmonary Regurgitation	5% (2/40)
**Average Blood values at discharge—Mean ± SD or Median [IQR]**	
Hemoglobin (g/L)	123 [114–135]
Sodium (mmol/L)	138 ± 4
Potassium (mmol/L)	4.3 [3.98–4.9]
Creatinine (µmol/L)	104 [80–120]
**Other heart failure medications received by patients**	
Beta Blockers	88% (35/40)
MRA	30% (12/40)
Diuretic	75% (30/40)
Diuretic dose—mg of Torasemide	
1–10	18
11–20	8
21–30	2
≥30	2
SGLT-2 inhibitors	27.5% (11/40)
**Patients with prior Heart Failure hospitalization during the year**	25% (10/40)
**Index hospitalization for heart failure**	65% (22/40)
**Frontline physicians for follow-up (absolute number—patients followed)**	
General Practitioner	80% (28/35)—29 patients
Cardiologist	20% (7/35)—11 patients

BMI—Body Mass Index, LVEF—Left Ventricular Ejection Fraction, MRA—Mineralocorticoid receptor antagonist, SGLT-2 inhibitors—Sodium-Glucose Transporter-2 inhibitors. Text in **bold** represents category titles.

**Table 2:** Questionnaire results.

Questionnaire Results	
Patients increased to ≥200 mg/day—%	25% (10/40)
Patients in whom an unsuccessful attempt to increase to ≥200 mg/day was made	10% (3/30)
Patients in whom physician attempted to increase to ≥200 mg/day but presented side effects that prevented the increase	100% (3/3)
Side effects presented by the patients in whom there was an attempt to increase to ≥200 mg/day	Symptomatic hypotension (3)
	Fatigue (1)
	AKI (1)
Reasons for which no attempt was made to increase the patient to ≥200 mg/day *	Titration was assumed to be cardiologist’s role: 56% (15/27)
	Pre-existing hypotension: 19% (5/27)
	Clinical stability: 15% (4/27)
	Lost to follow-up: 7% (2/27)
	Difficult follow-up: 4% (1/27)
	Other medication titration priorities: 4% (1/27)
	Potassium upper limit of normal: 4% (1/27)
	No specific reason provided: 4% (1/27)
	Previous failure to titrate: 4% (1/27)
	Double GP follow-up: 4% (1/27)
Physicians knowing target doses of S/V ^†^	65.7% (23/35)
Awareness concerning undemonstrated effect of S/V compared to Enalapril if S/V < 200 mg/day, in post hoc analysis of PARADIGM-HF	17.1% (6/35)

* Total > 100% because certain physicians provided multiple explanations for a single patient in some instances. ^†^ 35 physicians followed a total of 40 patients.

## Data Availability

The data presented in this study are available on request from the corresponding author due to restrictions related to subject privacy.

## Supplementary Materials

The following supporting information can be downloaded at: https://www.mdpi.com/article/10.3390/epidemiologia6030055/s1, Figure S1. Sacubitril/Valsartan titration algorithm Flowchart.

## Abbreviations

The following abbreviations are used in this manuscript:

ACE inhibitor	Angiotensin-converting enzyme inhibitor
ARNI	Angiotensin receptor-neprilysin inhibitor
ACC	American College of Cardiology
BMI	Body Mass Index
ED	Emergency Department
ESC	European Society of Cardiology
GDMT	Guideline-directed medical therapy
HFmrEF	Heart Failure with Mildly Reduced Ejection Fraction
HFpEF	Heart Failure with Preserved Ejection Fraction
HFrEF	Heart Failure with Reduced Ejection Fraction
ICER	Incremental Cost-Effectiveness Ratio
LVEF	Left Ventricular Ejection Fraction
MRA	Mineralocorticoid receptor antagonist
QALY	Quality-adjusted life year
SGLT-2 inhibitors	Sodium-Glucose Transporter-2 inhibitors
S/V	Sacubitril/Valsartan
