# Supplementary material for: Prescription Patterns of Sacubitril/Valsartan in an Outpatient Population Diagnosed with Heart Failure with Reduced Ejection Fraction After a Recent Hospitalization
Source: Epidemiologia (Basel). 2025 Sep 5;6(3):55. doi: 10.3390/epidemiologia6030055 (PMC12452757; doi:10.3390/epidemiologia6030055)
Supplement: Supplementary file 1 [file epidemiologia-06-00055-s001.zip › epidemiologia-3769307-supplementary.pdf]

Figure S1
Sacubitril/Valsartan titration algorithm

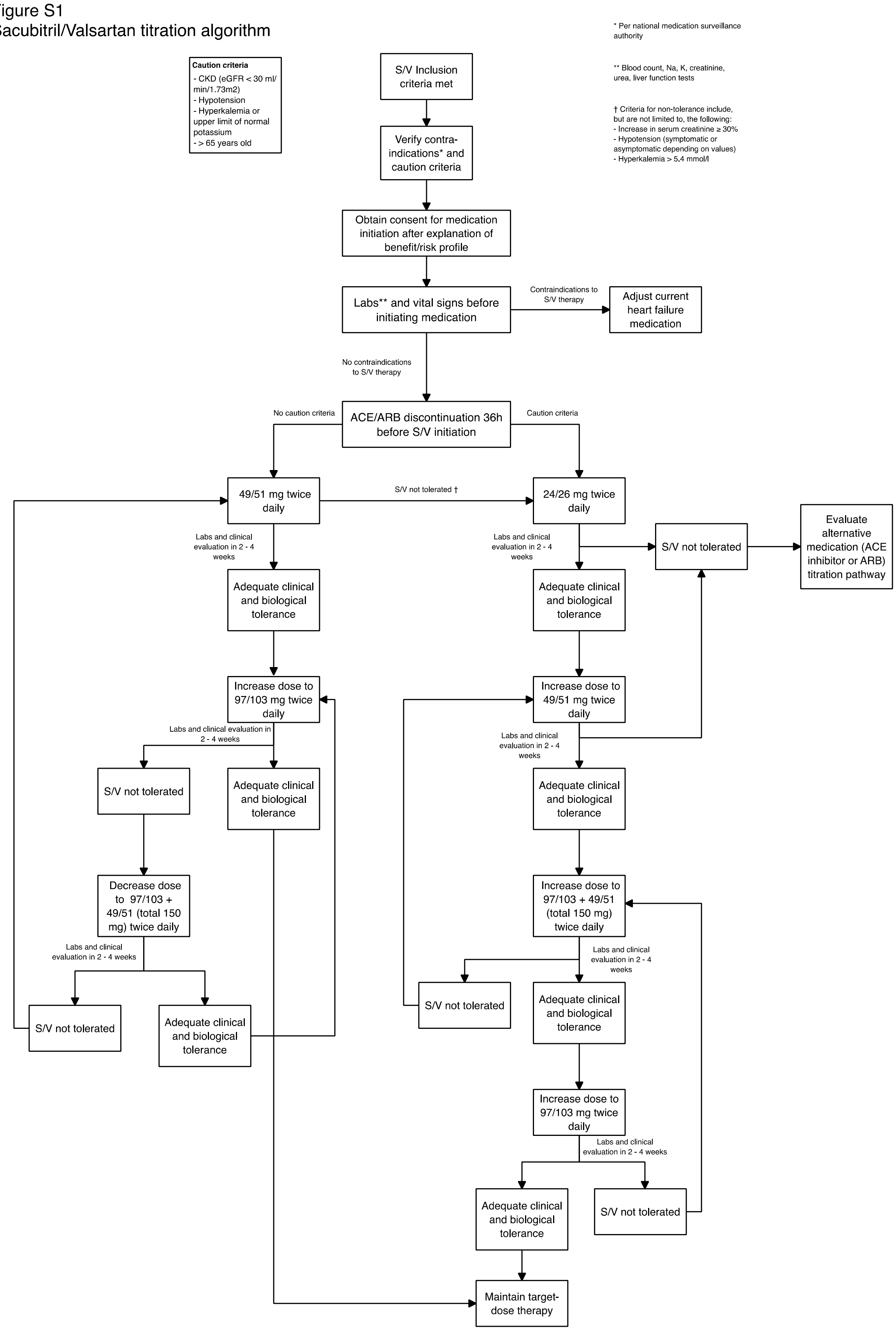

In the event where target doses cannot be achieved, but patient tolerates at least 49/51 twice daily: maintain highest tolerable dose with punctual increases in S/V dose to evaluate tolerability

Figure S1. Supplementary Material – Sacubitril/Valsartan titration algorithm.
